# A novel retrobulbar/intraconal surgical approach with combined cataract extraction and a modified AHMED ClearPath 250mm^2^ tube in the ciliary sulcus with an inferior sclerotomy in black and Afro-Latino patients with advanced glaucoma: a 6-month retrospective study

**DOI:** 10.3389/fopht.2026.1738832

**Published:** 2026-02-26

**Authors:** Daniel Laroche, Idaima Calderon, Imani Nwokeji

**Affiliations:** Advanced Eyecare of New York, City University of New York (CUNY) School of Medicine Department of Ophthalmology, New York University, New York, NY, United States

**Keywords:** advanced glaucoma, AHMED ClearPath glaucoma drainage device, combined cataract-glaucoma surgery, inferior sclerotomy, retrobulbar/intraconal placement

## Abstract

**Aim:**

The purpose of this early safety and technical feasibility study was to evaluate the real-world performance of cataract extraction combined with a modified Ahmed ClearPath 250 mm² glaucoma drainage device placed in the retrobulbar/intraconal space with inferior sclerotomy in glaucoma patients, focusing on surgical tolerability, device stability, and preliminary safety outcomes while acknowledging limitations in sample size and follow-up that preclude definitive efficacy claims.

**Methods:**

This study was conducted at Advanced Eye Care of New York, a private practice located in NY, NY. This was a single-center, retrospective study of 12 patients who underwent combined phacoemulsification cataract surgery and glaucoma surgery using a retrobulbar/intraconal AHMED^®^ ClearPath 250mm^2^ and an inferior sclerotomy technique. We report results at 6 months of follow-up. Investigated parameters were intraocular pressure, number of medications, mean deviation on visual field test, visual acuity, and adverse events.

**Results:**

Among all the 12 eyes undergoing combined cataract extraction and retrobulbar/intraconal AHMED^®^ ClearPath 250mm² placement with inferior sclerotomy, mean IOP decreased from 18.08 to 14.83 mmHg (18.0% reduction) at 6 months. Topical medications decreased from 2.67 to 1.0 (62.5% reduction). Visual field MD remained stable (-18.59 dB to -18.15 dB). Five patients achieved trabeculectomy-like results (IOP ≤12 mmHg on ≤1 medication). Complications were limited to temporary post-op hypotony and shallow anterior chamber, with no bleb, diplopia, or additional astigmatism.

**Conclusion:**

Cataract extraction combined with a modified Ahmed ClearPath 250 mm² glaucoma drainage device placed in the retrobulbar/intraconal space via inferior sclerotomy shows preliminary promise in reducing intraocular pressure and medication burden in Black and Afro-Latino patients with advanced glaucoma. These exploratory findings from a small cohort suggest technical feasibility and short-term safety, warranting further research with larger samples and longer follow-up to confirm efficacy and generalizability.

## Introduction

By 2050, primary open-angle glaucoma cases in the United States are projected to increase dramatically from 2.71 million to 7.32 million (1). The disease shows particularly concerning patterns among Black and Afro-Latino populations, who present with greater severity and faster progression compared to other demographic groups. African American patients face additional challenges, showing a higher risk of developing glaucoma at younger ages and experiencing more rapid disease advancement (1). These disparities are compounded by systemic inequities in healthcare access, affordability, and the availability of experienced ophthalmic surgeons (2). Elevated intraocular pressure (IOP) remains the only modifiable risk factor for glaucoma progression; however, traditional surgical approaches such as trabeculectomy are often associated with complications like bleb formation, hypotony, astigmatism, reduced visual acuity and the need for intensive postoperative care, which are challenges that are especially impactful in underserved communities (3, 4).

The enlarging lens further complicates glaucoma management, particularly in older patients, where lens thickening contributes to angle narrowing, pupillary block, and pigment dispersion, all of which increase resistance in the trabecular meshwork and elevate IOP (5). Although micro-invasive glaucoma surgeries (MIGS) offer safer alternatives with faster recovery and fewer complications, the high cost of devices like the Hydrus, iStent, Xen, and Preserflo, along with limited access in resource limited communities, reducing their real-world impact (6). More affordable options for MIGS, such as Sinskey hook goniotomy, needle goniotomy, and reusable Tanito Hooks, make this option available in resource poor areas around the world.

Trabeculectomy has been the gold standard and is quite affordable globally (7). However, trabeculectomy can fail and have sight-threatening complications such, blebitis, endophthalmitis.

The AHMED ClearPath 250 mm² glaucoma drainage device is a newer valveless implant designed to allow consistent posterior aqueous flow, with a configuration that is compatible with deeper anatomical placement and innovative surgical approaches. Its design theoretically offers greater intraocular pressure (IOP) reduction and lower medication burden, similar to other nonvalved implants, but bleb formation, encapsulation, and failure remain major limitations that justify exploration of alternative placement strategies in an effort to improve long-term outcomes. In the broader context of glaucoma surgery, standard ClearPath placement shares many of the complications seen with traditional tube shunts: Ahmed valved implants show declining long-term success (from about 89% at 6 months to roughly 26% by 15 years) with high rates of bleb encapsulation and hypertensive phases that frequently require secondary interventions (8).

Valveless have demonstrated superior long-term IOP control compared with valved devices but typically require flow restriction techniques (e.g., ligatures, intraluminal stents) to reduce early hypotony risk, which adds surgical complexity that the ClearPath design seeks to streamline. Nonetheless, encapsulation remains the leading cause of failure across all tube designs, with histopathology showing thick fibrotic capsules that impede flow and anterior segment OCT confirming that greater bleb wall thickness correlates with surgical failure, regardless of valve mechanism (9). Because conventional superotemporal or superonasal placement exposes the plate to similar conjunctival and episcleral scarring responses, deeper or less traumatized anatomical planes such as retrobulbar or intraconal locations are being investigated to reduce conjunctival scarring, lower exposure risk, and potentially modify the fibrotic capsule pattern, addressing a fundamental limitation common to all current tube shunt approaches.

This study explores a novel approach involving the retrobulbar/intraconal placement of a modified AHMED ClearPath 250mm² device in conjunction with an inferior prophylactic sclerotomy combined with cataract extraction, in Black and Afro-Latino patients with advanced glaucoma. The inferior sclerotomy was made to serve as a drain for any choroidal effusion that may occur since this can be as high as 30% when aiming for single digit 8-9mmHG intraocular pressures post-operatively.

In this single-center, 6-month retrospective pilot study, we evaluated real-world surgical outcomes, including IOP reduction, medication burden, visual field stability, and postoperative safety in this high-risk demographic. Our findings demonstrate that this modified technique may offer a safer, effective, and scalable solution to address both the clinical and socioeconomic challenges faced by Black and Afro-Latino patients with advanced glaucoma.

## Methods

This was a single-center, retrospective pilot study evaluating Black and Afro-Latino patients (self-identified corresponding with the local community makeup) with advanced glaucoma who underwent a novel combined surgical approach at Advanced Eye Care of New York, a private ophthalmology practice located in Harlem, New York City.

Patients underwent simultaneous phacoemulsification cataract extraction and implantation of a modified AHMED^®^ ClearPath 250mm² glaucoma drainage device (New World Medical, Rancho Cucamonga, CA, USA) placed in the retrobulbar/intraconal space, accompanied by an inferior sclerotomy. All patients who underwent this procedure between April 2024 and October 2024 and completed 6 6-month follow-ups. The modified technique was designed to reduce intraocular pressure (IOP) without the formation of a filtering bleb and to minimize anterior segment complications such as diplopia or induced astigmatism.

A total of 12 eyes from 12 consecutive patients with no previous incisional eye surgery were included. All patients identified as Black or Afro-Latino and had visually significant cataracts and advanced open-angle glaucoma, with insufficient IOP control despite maximal tolerated topical therapy. Data was collected preoperatively and postoperatively on day 1, month 1, and month 6.

The data collection focused on key clinical parameters that reflect both efficacy and safety, including intraocular pressure (IOP), number of glaucoma medications, visual field mean deviation (MD), and best-corrected visual acuity. Additional attention was paid to adverse postoperative events, such as hypotony, shallow anterior chamber, bleb formation, diplopia, and induced astigmatism. This comprehensive set of variables allowed for a multidimensional assessment of the outcomes associated with this novel surgical technique, providing a detailed evaluation of pressure-lowering effectiveness, functional vision preservation, medication reduction, and postoperative safety in this high-risk population. Descriptive statistics and observational data characterized the outcomes in this study.

This study was conducted following the ethical standards of the Icahn School of Medicine Institutional Review Board. A waiver of informed consent was approved by the New York Eye and Ear Infirmary of Mount Sinai Institutional Review Board (IRB- STUDY-23-00815) due to the retrospective nature of the study. This study was conducted in accordance with the ethical principles outlined in the Declaration of Helsinki.

### Procedure

The primary goal of surgery was to achieve a meaningful reduction in IOP and medication burden while preserving visual function. The AHMED ClearPath 250 device was trimmed with the rounded sides removed. The posterior plate was trimmed to a distance to fit along the globe between the equator and the optic nerve to minimize interference with intraconal retrobulbar structures.

The rounded sides of the Clearpath 250 were trimmed. A novel Laroche retrobulbar tube equation was used to calculate the length of the tube, and the plate should be:

Retrobulbar tube length (to fit between the equator and optic nerve mm = (π × (1/2 axial length of the eye)) − 3

**Figure 1:**
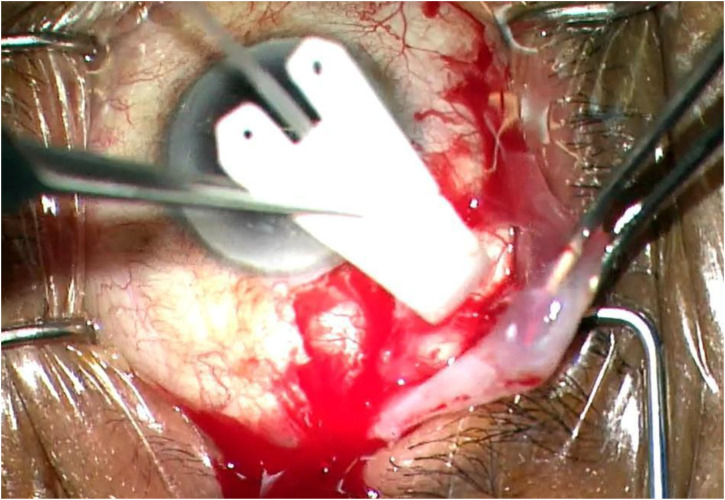
Modified Ahmed Clearpath 250 being inserted into the retrobulbar space.

**Figure 2:**
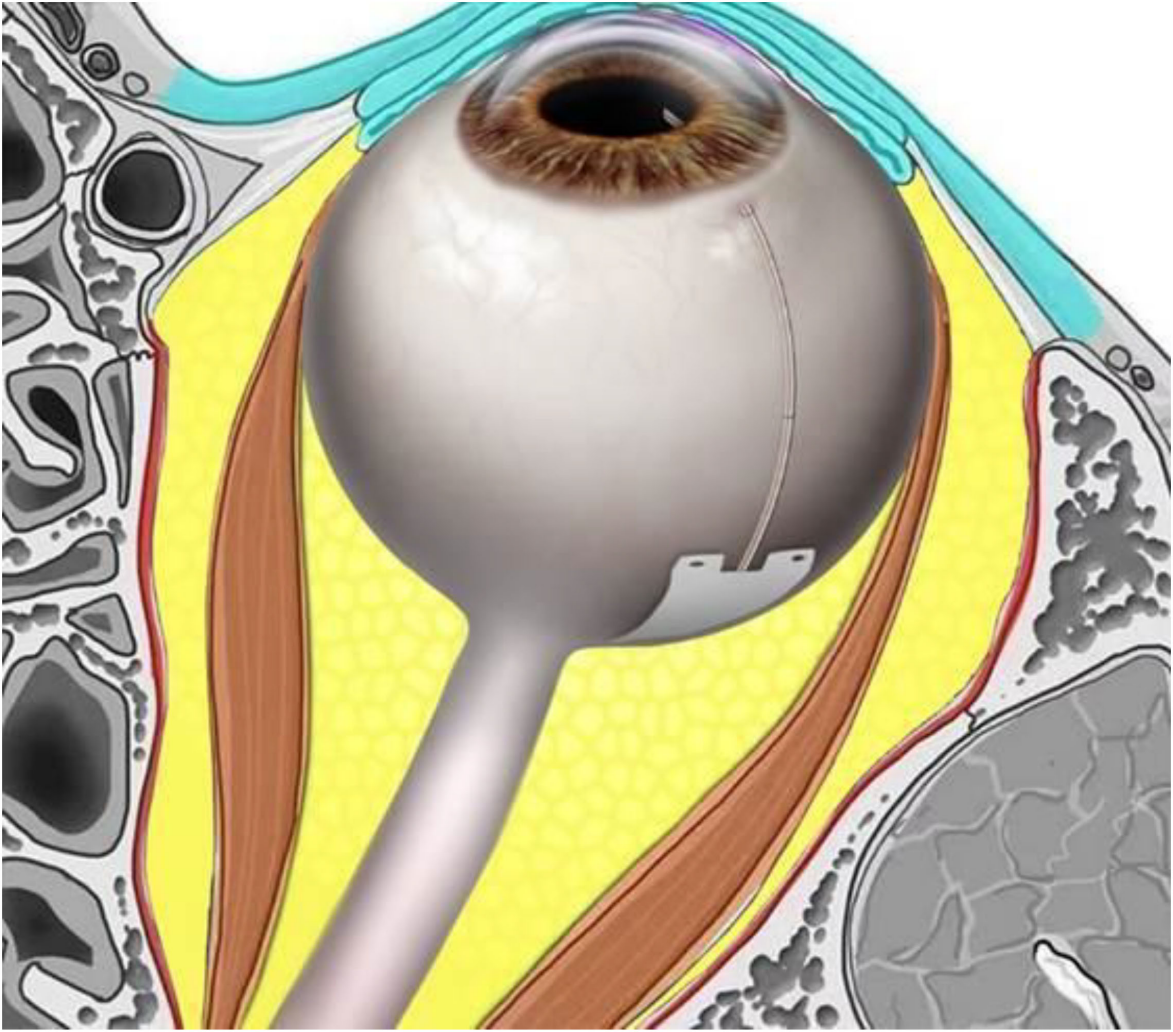
Schematic image of eye with optimal position of plate location in the retrobulbar intraconal spaced between the equator and optic nerve with the Laroche retrobulbar length equation. (Length in mm= pi x (1/2 axial length) -3.

The posterior end of the Ahmed Clearpath 250 plate was cut prior to implantation in the retrobulbar/intraconal space to facilitate posterior aqueous drainage and not interfere with other structures in this space (**Figures 1**, **2**).

**Figure 3:**
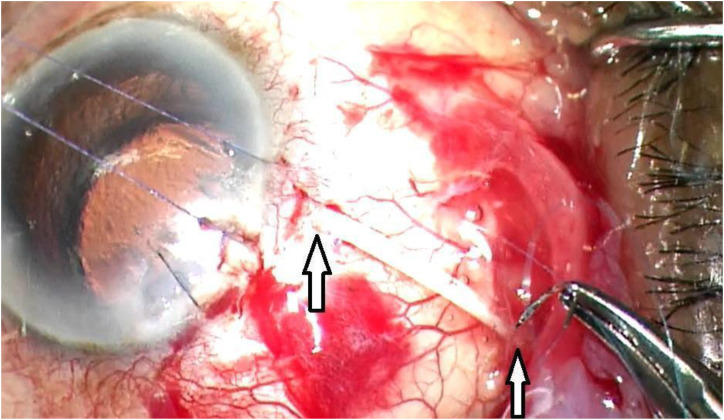
Two points of fixation with 10-0 prolene suture to secure the tube to sclera.

**Figure 4:**
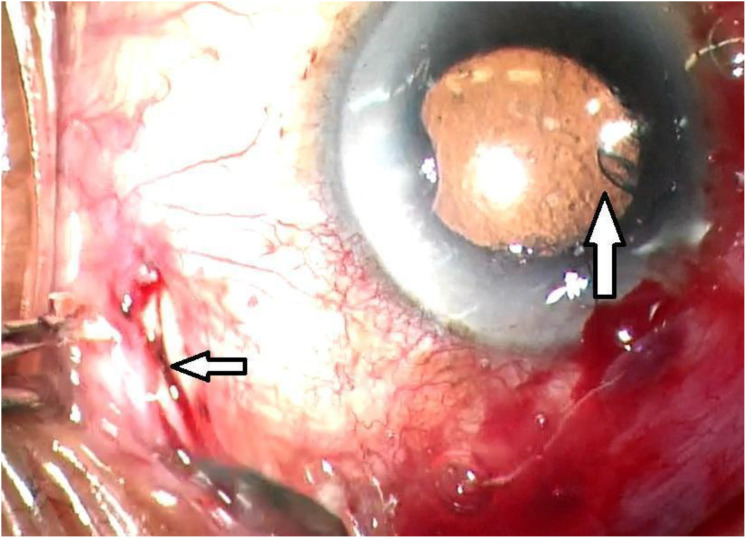
Superior arrow pointing to the tube in the ciliary sulcus, inferior arrow pointing to inferior scleral window.

The patients underwent informed consent. The patient was prepped and draped in the usual sterile manner for the ophthalmic procedure. The surgeon (DL) sat temporally. Topical lidocaine gel was placed. Klott forceps and Westcott scissors were used to perform a superotemporal conjunctival peritomy. A peribulbar block of 4 cc of 2% preservative-free lidocaine and 0.75% Marcaine was given. The modified Ahmed Clearpath 250 tube and plate were then measured and cut to the individualized length with the Laroche equation and then inserted into the retrobulbar/intraconal space above tenon’s capsule. The anterior tip of the tube was cut bevel down. Traditional, clear, uncomplicated cornea cataract surgery was performed in the usual manner temporally. It is important to have a 5mm capsulorhexis and place the intraocular lens into the capsular bag to assist in maintaining the anterior chamber postoperatively. A suture was used the close the temporal clear cornea cataract surgery wound the maintain the anterior chamber during insertion of the tube. Healon was inserted through a paracentesis to create a space between the posterior iris and the intraocular lens. A caliper was used the measure and mark, 1.5 mm from the limbus with ink. A 23-gauge needle was used to create an opening into the ciliary sulcus. The tube was then inserted with two.12 forceps into the ciliary sulcus approximately ½ way between the limbus and the center of the optical axis of the intraocular lens. The tube was then secured to the sclera with a 10-0 prolene suture through the tube and the sclera near the entry into the ciliary sulcus. A second 10-0 prolene suture was used to secure the tube to the sclera approximately 10mm posteriorly or as far as visibly possible towards the equator (**Figure 3**). The tube was then covered with a cornea-scleral patch (Katena, large). The patch was sutured with interrupted 8-0 vicryl sutures. The conjunctiva was then closed with 8-0 vicryl sutures in a running locking manner. An inferior conjunctival dissection was performed and a prophylactic sclerotomy was performed 4.5 mm from the limbus with.12 forceps, a crescent blade, and Westcott scissors, since when aiming for low IOP of 8-12 mmHg, there is a high risk of choroidal effusions of about 30%. This prophylactic sclerotomy reduces the risk and need for an intractable choroidal effusion and need for return to the operating room (**Figure 4**).

Adjustments to each patient’s glaucoma medication regimen were made during the follow-up period based on clinical judgment, IOP levels, and ocular examination findings. The primary outcome measure was IOP reduction compared to baseline. Secondary outcomes included a reduction in the number of glaucoma medications, visual field mean deviation (MD), best-corrected visual acuity, postoperative complications, and refractive outcomes. The safety profile was assessed by monitoring for adverse events such as postoperative hypotony, shallow anterior chamber, bleb formation, diplopia, and induced astigmatism.

## Results

Twelve eyes from twelve patients underwent combined phacoemulsification cataract extraction and implantation of a modified AHMED^®^ ClearPath 250mm² glaucoma drainage device placed into the retrobulbar/intraconal space with an inferior sclerotomy. All patients identified as Black or Afro-Latino and had advanced glaucoma with visually significant cataracts based on visual acuity and visual field loss. Subsequently, data was analyzed at the 6-month follow-up mark.

At baseline, the mean age was 68.9, intraocular pressure (IOP) was 18.08 mmHg. There were 6 males and 6 females. Ocular hypotensive medications were.2.67. There was no medication washout. The baseline LogMAR was 0.53 ant baseline MD on the visual field was -18.59. By the 6-month postoperative visit, the mean IOP had decreased to 14.83 mmHg, representing an 18.0% reduction in pressure across the cohort. In addition to IOP reduction, patients experienced a significant reduction in medication burden. The average number of topical ocular hypotensive medications decreased to 1.0 per patient, an overall reduction of 62.5% (**Table 1**).

**TABLE 1:** Pre and post-operative IOP, medications and visual field.

Parameter	Baseline (Pre-op)	6 Months Post-op	% Change	Notes
Mean Intraocular Pressure (IOP)	18.08 mmHg	14.83 mmHg	↓ 18.0%	Significant reduction in IOP across the cohort
Topical Medications	2.67	1.0	↓ 62.5%	Marked reduction in medication burden
Visual Field (Mean Deviation)	–18.59 dB	–18.15 dB	Stable	No measurable progression in visual field loss

In this cohort of advanced patients with central vision loss, visual acuity improved from LogMAR 0.53 to 0.46, representing a significant gain of approximately 7 letters (or 0.07 log units). This improvement corresponds to a shift from poorer vision (around 20/80) toward better visual function (approximately 20/50).Visual field stability was maintained throughout the follow-up period. The mean deviation (MD) on visual field testing remained essentially unchanged, with a postoperative value of –18.15 dB at 6 months, indicating that no measurable visual field progression occurred during the study period.

Furthermore, five of the twelve patients (41.7%) achieved surgical outcomes comparable to trabeculectomy, defined as achieving an IOP of 7-12 mmHg or lower while using no medications at 6 months postoperatively. The procedure demonstrated a beneficial safety profile. Postoperative complications were limited to six cases of temporary hypotony accompanied by a shallow anterior chamber requiring healon injection within the first week. These events were resolved without the need for reoperation or long-term sequelae. There were no cases of bleb formation, diplopia, induced astigmatism, or other sight-threatening complications. All patients maintained or improved their best-corrected visual acuity, and no further surgical interventions were required during the 6-month follow-up period.

## Discussion

This retrospective pilot study evaluated a novel combined surgical approach in Black and Afro-Latino patients with advanced open-angle glaucoma. The technique integrates phacoemulsification cataract removal with strategic placement of a modified Ahmed ClearPath 250 mm² drainage device in the retrobulbar/intraconal space, deep within the muscle cone behind the eye (**Figure 2**). This creates a direct aqueous pathway from the anterior chamber to the orbit, bypassing damaged anterior structures like the trabecular meshwork, Schlemm’s canal, and collector channels. A prophylactic inferior sclerotomy provides drainage for potential choroidal effusions during intraocular pressure (IOP) reduction. Bleb-independent by design, the posterior positioning leverages the orbit’s natural negative pressure gradient typically 4-6 mmHg for sustained fluid absorption.

**Figure 5:**
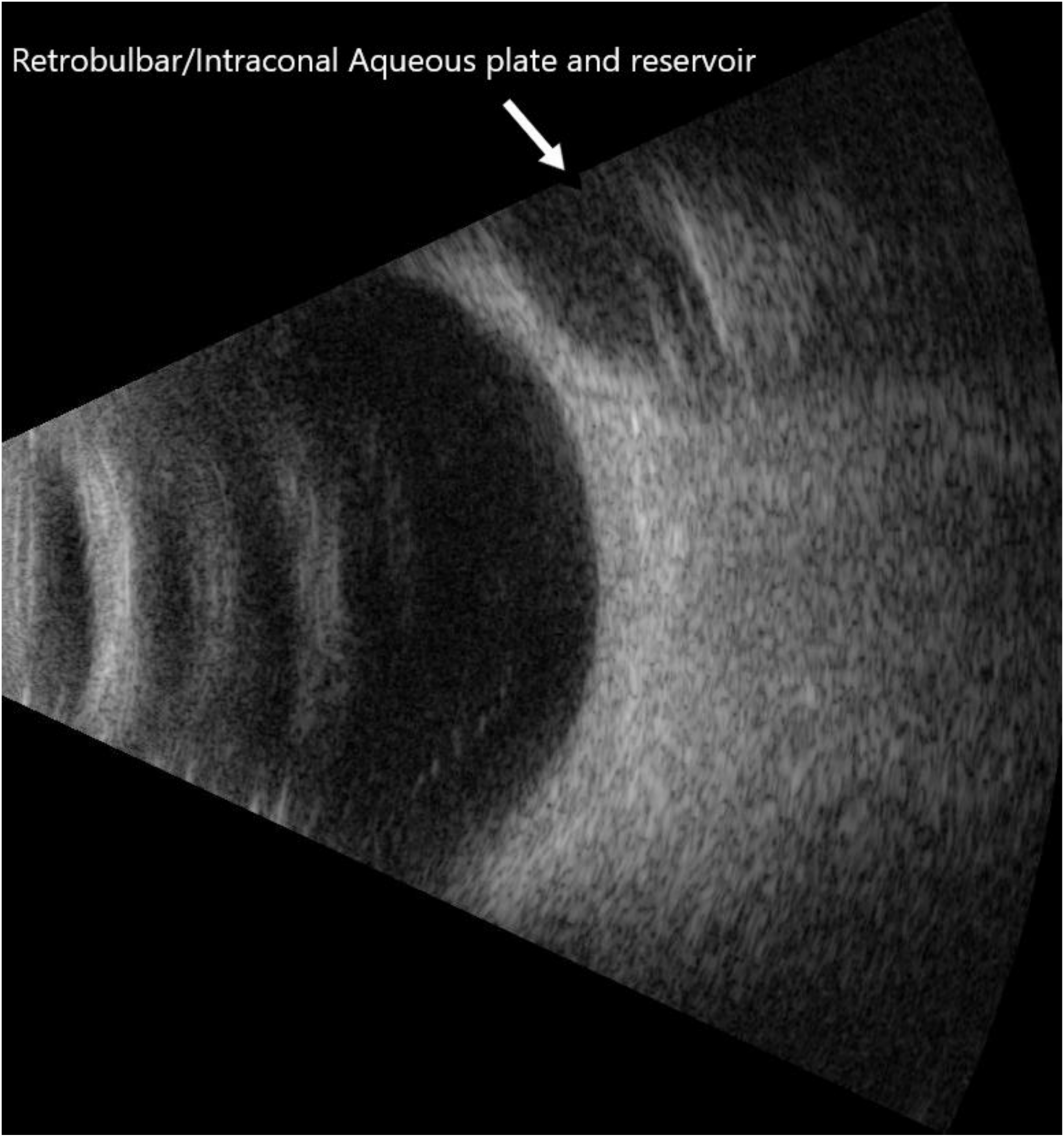
Bscan of retrobulbar/intraconal aqueous reservoir and plate.

Excision of Tenon’s capsule with supra Tenon’s plate placement minimizes encapsulation and failure, eliminating the need for antimetabolites that risk hypotony, toxicity, and necrosis in traditional surgeries. Orbital pressure dynamics, influenced by fat, connective tissues, blood vessels, extraocular muscles, and comorbidities like hypertension or diabetes, support low-target IOPs in the single digits without fibrotic interference, as the retrobulbar/intraconal space lacks Tenon’s or conjunctival barriers (**Figure 5**). Caution applies in low-aqueous states (e.g., ocular ischemic syndrome, diode cyclophotocoagulation, or periorbital radiation), where hypotony risk rises. The learning curve for the retrobulbar intraconal technique is relatively short for experienced surgeons, as the procedure largely mirrors standard approaches. The key modifications include performing a tenenectomy, extending the dissection more posteriorly, positioning the plate deeper beneath Tenon’s capsule, and securing it by suturing through the tube rather than the eyelets. Beyond these adjustments, the overall surgical steps remain quite similar. All eyes were done with this technique.

Early hypotony in initial cases stemmed from short-acting Healon (sodium hyaluronate 1%); switching to Viscoat (sodium hyaluronate 3%-chondroitin sulfate 4%) can markedly reduced shallow chambers and reinjections, mirroring comparative data showing Viscoat’s superiority (hypotony: 8.3% vs. 40.7%; shallowing: 8.3% vs. 37%). In-the-bag IOL with 5 mm capsulorhexis adds posterior support prevents shallowing in the anterior chamber. We currently have started using the Ahmed ClearPath 250 ST—featuring a narrower tube (ID 127 μm vs. 305 μm; OD 457 μm vs. 635 μm; 6 mm longer for intraconal reach) enhances flow resistance via 25-gauge needle insertion and 6-0 Prolene ripcord, curbing over-filtration without ligation to help reduce hypotony. The Ahmed FP7 offers a valved alternative to help reduce hypotony.

The retrobulbar space divides into intraconal (optic nerve, ophthalmic artery/vein, cranial nerves III/IV/VI amid fat cushioning) (10–14) and extraconal (lacrimal gland/sac, veins, nerves, fat) compartments; the Laroche retobulbar equation ensures safe tube length beyond the equator, avoiding structures. Over 6 months, outcomes included 18% IOP reduction, 62.5% medication cut, and 41.7% trabeculectomy-like success (IOP ≤12 mmHg off meds), preserving visual fields (-18.59 to -18.15 dB) and acuity. Transient hypotony/shallowing affected six eyes (resolved post-Healon), with no diplopia, bleb issues, astigmatism, exposures, or reoperations—advantages for scarred conjunctiva, inflammation, diabetes, immunosuppression, or prior surgeries.

The modest baseline IOP likely reflects the absence of a preoperative medication washout. While cataract surgery alone can reduce IOP by approximately 2–3 mmHg or to the mid-teens, it typically does not achieve the lower pressures of 8–12 mmHg often required in patients with advanced glaucoma.

This modified surgical technique can directly address the well-documented challenges in managing glaucoma in Black and Afro-Latino patient populations facing aggressive disease, poor traditional outcomes, and barriers to follow-up/medication adherence. Ciliary sulcus placement and posterior strategy slash infection/exposure risks (15). Limitations of this study include the small sample size (n=12), retrospective design, limited 6-month follow-up period, requirement for specialized surgical expertise, variability in Tenon’s capsule anatomy, absence of control groups, and descriptive statistics due to the small cohort size. Retrobulbar risks (motility, proptosis, infection) warrant consent; encapsulation could prompt quadrant revision or repeat surgery in another quadrant.

These preliminary results position cataract extraction with retrobulbar/intraconal Ahmed ClearPath 250 (or equivalents like Aurolab, Ahmed FP7 etc…) (16) and sclerotomy as a safe, effective option yielding filtration-level IOP control without bleb drawbacks, ideal for advanced glaucoma cases and potentially post-failed trabeculectomy/tubes. Larger prospective, multi-institutional trials with long-term data, controls, cost analyses, and patient-reported outcomes are essential to affirm efficacy, safety, and scalability in diverse settings.

## Data Availability

The original contributions presented in the study are included in the article/supplementary material. Further inquiries can be directed to the corresponding authors.
